# ‘Eh! I felt I was sabotaged!’: facilitators’ understandings of success in a participatory HIV and IPV prevention intervention in urban South Africa

**DOI:** 10.1093/her/cyv059

**Published:** 2015-11-19

**Authors:** Andrew Gibbs, Samantha Willan, Nwabisa Jama-Shai, Laura Washington, Rachel Jewkes

**Affiliations:** ^1^Health Economics and HIV/AIDS Research Division (HEARD), University of KwaZulu-Natal, South Africa,; ^2^Gender and Health Research Unit, Medical Research Council, Pretoria, South Africa, and; ^3^Project Empower, Durban, South Africa

## Abstract

Participatory approaches to behaviour change dominate HIV- and intimate partner violence prevention interventions. Research has identified multiple challenges in the delivery of these. In this article, we focus on how facilitators conceptualize successful facilitation and how these understandings may undermine dialogue and critical consciousness, through a case study of facilitators engaged in the delivery of Stepping Stones and Creating Futures and ten focus-group discussions held with facilitators. All facilitators continually emphasized the importance of discussion and active engagement by participants. However, other understandings of successful facilitation also emerged, including group management—particularly securing high levels of attendance; ensuring answers provided by participants were ‘right’; being active facilitators; and achieving behaviour change. These in various ways potentially undermined dialogue and the emergence of critical thinking. We locate these different understandings of success as located in the wider context of conceptualizations of autonomy and structure; historical experiences of work and education; and the ongoing tension between the requirements of rigorous research and those of participatory interventions. We suggest a new approach to training and support for facilitators is required if participatory interventions are to be delivered at scale, as they must be.

## Introduction

Behavioural interventions to prevent the sexual transmission of HIV and intimate partner violence (IPV) often adopt group-based methodologies of behaviour change that seek to support the development of critical consciousness amongst participants [1–7]. These draw on Freire’s [8] theorization of critical consciousness as their underpinning theory of behaviour change as developed by social psychologists [7, 9, 10]. Freire [8] argues that there are two models of education for change. The first is education as banking, whereby knowledgeable educators transfer information to students, in didactic processes [8]. Students learn from these educators and knowledge is ‘built up’ within them. Numerous studies have outlined how didactic approaches to behaviour change have had little impact, particularly in contexts of high inequality [11–13].

The second educational approach situates the educator as a facilitator [8]. In this understanding the facilitator assumes participants are knowledgeable about their own lives and primarily need facilitators to create a safe social space and to support participants to engage in dialogue about issues of importance in their own lives [11, 13]. Through dialogue in a safe social space and supported by a facilitator, who prompts and questions the participants, it is assumed participants will develop critical thinking—starting to recognize the underlying factors shaping their behaviour, essentially moving from identifying risk factors for a health issue, to the factors that shape broader vulnerability [9, 14]. At its most radical, this approach assumes all necessary knowledge on a topic exists within a group and the role of the facilitator is to merely prompt questions and support learning rather than introduce ‘new’ information [15].

The educator as facilitator approach is seen to have a number of positive outcomes. It allows participants to translate new knowledge into their everyday vernacular and experiences and integrate this into their actions [7, 13]. At a broader level participants can start to develop new identities that are health enhancing, which stand in opposition to previous identities that are linked to health compromising behaviours [7]. Success therefore in participatory, facilitator led health interventions is seen through facilitators being able to support participants to engage in discussion on relevant topics, which generates critical thinking, enabling participants to develop new understandings about themselves and the world they live in and eventually act in ways that support improvements in their health.

While there are clear assumptions about the roots of successful facilitation, literature outlines the challenges in translating these into real-world contexts. HIV-prevention research highlights how the ideals of facilitation disappears in the actual delivery of interventions and facilitators move towards didactic approaches of education [14, 16, 17]; central to this has been that most facilitators’ historical experiences of education have been didactic and they have struggled to operationalize a different form of learning [16, 18]. Moreover, facilitators often prefer to draw on biomedical frameworks to explain HIV, with their relatively clear and ‘correct’ answers, as opposed to supporting participants to develop social understandings of health, which often have less clearly ‘correct’ answers, although these are the ones that will support reflection on the wider drivers of vulnerability and behaviour [16].

Research also identifies how facilitators struggle to operationalize concepts and ideas—often introduced to them in English—into local language and concepts [19, 20]. This highlights the on-going issue of the ‘importing’ of interventions into local contexts [13].

Moving away from the narrow confines of facilitated spaces recent work has pointed to the importance of wider social contexts in shaping the potential for dialogue and action [21]. Factors such as poverty and gender inequalities limit the potential for open conversations and the ability of young people to translate new ideas and concepts into action [16, 21–23].

Despite extensive research, there is little understanding of how facilitators conceptualize what ‘good’ facilitation is. Shifting the focus to facilitators’ own conceptualizations of successful facilitation provides an opportunity to reflect on how they understand theoretical concepts when put into practice and factors shaping this. Using a case study of facilitators’ implementing a participatory intervention targeting HIV-risk behaviours and IPV in young people in urban informal settlements in South Africa, we seek to understand how facilitators conceptualized successful facilitation.

## Materials and methods

### Intervention

We undertook a feasibility study of a participatory, facilitator-led intervention—Stepping Stones and Creating Futures—that sought to reduce HIV-risk behaviours and IPV amongst young women and men (18–30) in urban informal settlements in South Africa. The intervention for participants comprises 21 group sessions, each 3 h long and primarily single sex, led by a trained facilitator and delivered over about 12 weeks, i.e. two sessions per week [24, 25]. Sessions encourage participants to reflect on gendered norms, communication, HIV, contraception and violence, as well as strengthen their livelihoods, through critical thinking about futures and making plans for income generation based on a realistic assessment of available resources. Sessions draw on adult education theories, in particular Freire [8], and use participatory techniques such as drama, discussion, participatory mapping and reflective diaries (journaling) to encourage discussion, reflection and critical thinking [26–28].

The pilot intervention was delivered by six facilitators (three female, three male) over 12 weeks. All had some previous experience of facilitation, ranging from running focus-group discussions through to group-facilitation. One facilitator came from the community in which the intervention ran, although he was slightly older and financially better off than participants. The other five were not from the same community and were slightly better off than participants, some had post-secondary education or training. Despite their relative affluence compared to participants, the facilitators did not have fulltime work (until employed by the project) and continued to struggle financially, following the completion of the intervention.

Recognising many peer-led interventions fail because of a lack of training and support [12, 16] facilitators received 5 weeks of training and on-going support throughout the pilot to promote participatory approaches. The training was 2 weeks of experiencing the intervention as participants; 2 weeks of additional content training e.g. on HIV-transmission, contraception, as well as sessions on facilitation skills and a final week practicing facilitation. Throughout the intervention a senior trainer was available for facilitators to discuss issues they faced. In addition, weekly meetings amongst facilitators, the senior trainer and the research team, provided opportunities for the senior trainer to role-model ‘good’ facilitation during debrief sessions, through probing facilitators to think through the problems they faced and discuss them as a group.

Study outcome and process evaluations have been published elsewhere; in summary they show that at 12 months women reported less sexual and/or physical IPV in the past 3 months (37% *P* = 0.037), and women’s and men’s gender attitudes improved significantly and men’s relationship control became more equitable [29]. The intervention improved livelihoods, with increases in earnings and more people seeking work [29]. Qualitative data on men’s experiences emphasized how the intervention created safe social spaces for men to talk about the challenges they faced as young men living in urban informal settlements [5]. They particularly liked this as they felt they did not have these spaces in their everyday lives. It also emphasized the wider contextual challenges they experienced in attempting to act on the new identities and opportunities they identified [5, 22].

### Data

Data come from 10 team meetings with facilitators (*n* = 6), the senior trainer who supervised and supported facilitators, and the first author of this article. These weekly meetings occurred during the implementation of the intervention and sought to reflect on the past week and the challenges facilitators faced. Team meetings lasted between 45 min and 3 h, averaging 2 h and were led by the senior trainer. Team meetings focused on the facilitators’ experiences of running sessions, discussing challenges faced and trying to promote participatory approaches. There was no topic guide or attempt to shape the discussions of the team meetings, rather the discussions were shaped by the facilitators’ own priorities. Data were collected as part of a wider process evaluation of the project.

Team meetings were digitally recorded and were conducted in a mix of English and isiZulu. A research assistant translated and transcribed these. Data analysis was conducted using thematic network analysis, using open coding focused on contrasting understandings of success [30, 31]. Themes identified are presented below.

Ethical approval for the study was given by the University of KwaZulu-Natal (HSS/0789/011 and HSS/1273/011D) and the South African Medical Research Council (EC003-2/2012). All participants provided written informed consent and were aware that the team meetings were being used as part of the wider research project.

## Results

Data suggest facilitators held contrasting views on what constituted successful facilitation. All facilitators explicitly recognized that success in facilitation meant that they had supported participants to be active and engage in dialogue—all important in underpinning the emergence of critical thinking. Yet in FGDs facilitators also described other forms of success, including group management—primarily around ensuring attendance and punctuality of participants—facilitators leading groups and making sure ‘correct’ and ‘good’ answers were provided and ensuring behaviour change happened. These different understandings of success emerged through the ongoing daily management of the intervention and the expectations placed on facilitators, as well as the wider context in which the intervention was delivered, and potentially undermined the emergence of critical thinking in the group.

### Discussion and active engagement by participants

Participatory approaches to behaviour change are premised on the assumption that through the creation of safe social spaces and supporting discussion amongst participants group processes leading to reflection, critical thinking and change [9, 11, 13]. Throughout the training and the intervention a significant effort was made to encourage facilitators to recognize the importance of this sort of participatory experience for young people. This ongoing engagement and emphasising paid dividends, with a key theme emerging from the data being that successful sessions were ones in which participants had engaged in discussion:

Wanda (male): But ya, they are grasping everything that we are learning in class and there is a high level of maximum participation and discussion, and I guess in this point in time they are just showing they are now relaxed with each other and they can relate to one another’s feelings and, ya, because there is so much respect in the room.

Of the 21 sessions in the intervention only one of the sessions mixed women and men together and this came towards the end of the intervention. The assumption was it would allow women the space collectively to build their confidence to speak openly in front of men, who they often deferred to in other social situations [32]. Reflecting on this mixed sex session, facilitators highlighted how active women and men had been in engaging in dialogue between each other:

Mamuntu (female): We had a nice meeting on Wednesday. The group was quite big, which is very surprising. So we ended up with thirty people in the group. And the levels of participation were just amazing, like they were very on to it.

Jabu (male): To add on to what Mamuntu was saying, it was great working with Mamuntu. She was dedicated. The participation was high. People were excited. People were interacting. Everything was in a high level and in a high standard.

In all the discussions facilitators recognized the importance of promoting dialogue and discussion as being central to a successful intervention and sought to achieve this throughout sessions.

### Group management

Overall attendance at sessions by participants was ∼60% [29]. Qualitative research explored barriers to attendance identifying a range of factors including accessing the minibus taxi fare—which was reimbursed on a daily basis—the continual need to search for a job to survive and for some young women how their male partners simply refused to let them attend [22]. In this context of often erratic attendance by some participants, another central theme of success for facilitators was around group management—specifically ensuring participants’ high levels of attendance at sessions and prompt arrival of participants. For facilitators these two factors became a marker of success. As Xoli commented after one particularly challenging week:

Xoli (male): This week we started badly when we suffered a major blow because of the poor attendance from the guys. Eh, I felt I was sabotaged!

Facilitators were highly invested in an understanding of success reflected in high levels of attendance at sessions. Facilitators tried different techniques to encourage greater attendance and punctuality, through working with participants. This included getting participants to agree start times for sessions, regular reminder phone calls to participants and delaying the start of sessions when participants did not arrive. Yet these did not work to improve attendance or punctuality and facilitators grew increasingly frustrated:

Thandi (female): Eh, *(laugh)* I don’t know where to start seriously because I have run out of strategies and I realize that I am getting really pissed off with that group. On Monday we were finishing Stepping Stones and the first challenge is that some people won’t come for three sessions, they come on the fourth session and be all lost and cocky about it and then not come for three after that.

Increasingly the project team also recognized this as a major challenge to the intervention and potentially undermining project outcomes. To encourage facilitators to follow-up on participants the team offered small rewards to facilitators based on who had called the most participants to remind them or who had most participants at sessions.

At the same time facilitators started to move beyond simply being frustrated to positioning young people in particular problematic ways. Wanda described the young men as being lazy:

Wanda (male): With my Monday group, yo! Eh! Half of them are very early and half of them are very lazy. The reason why I am saying they are lazy, they are lazy in the context that they can’t tell me in advance they won’t make it or they are having trouble with the money or whatever.

This conceptualization of successful facilitation focused on group management led facilitators away from focusing on supporting participation and dialogue in the group towards a more structured understanding of success as being on attendance and punctuality, potentially undermining their emphasis on dialogue and critical reflection. Moreover, facilitators began to position young people as lazy, rather than recognising the significant structural constraints they lived under. Through positioning young people as lazy facilitators inadvertently reinforced young people’s social marginalization and undermined the potential for critical thinking to emerge in the group [33].

### Getting answers ‘right’

Participatory approaches to behaviour change assume participants are ‘experts’ in their own lives and have all the knowledge and information they require, based on their lived experiences [8, 15]. Such an understanding requires facilitators to reposition themselves away from being ‘experts’ who provide correct answers to any question to playing a role of questioning, probing and supporting participants to come to understand the world they live in [9, 21]. Repositioning oneself away from the expert to someone who supports the development of knowledge is challenging, particularly in contexts where the overwhelming experiences of education have been didactic and ‘top-down’ as for the facilitators in South Africa [13, 18].

The facilitators described how they felt conflicted when listening to participants’ answers to questions or watching the dramas that they had created and facilitators felt participants had not provided the ‘correct’ answers. Jabu, for instance, described watching a participant developed sketch on how participants would respond to seeing a young man hitting his girlfriend in the street—their response was to do nothing. Jabu got frustrated with this response and demanded that they recreate the drama with them intervening. Yet in doing this in a demanding and didactic way, Jabu missed an opportunity to open up a discussion with young men about why they felt they could not offer a response and support them to see this as a collection of factors including: the lack of police systems, widespread acceptance of violence against women and what options they could envisage. Rather Jabu felt they simply had not tried hard enough to imagine a solution:

Jabu (male): I don’t know whether I am right or wrong but some of the answers I tell them, that, ‘here you were supposed to say like that.’ … Sometimes I tell them that I feel like you didn’t put 100% to, especially role plays. I tell them, ‘if maybe you can try another example of this and that, maybe you can come up with the good results rather than this example you did.’

For facilitators whose only experience of education had been one of ‘banking education’ [8], developing alternative ways of interacting with participants proved challenging, particularly as the facilitators were positioned as ‘experts’ in these groups. Through providing what they felt to be correct answers, rather than enabling participants to come to their own understandings of the social world, facilitators may have foreclosed dialogue and critical thinking in the intervention.

### Being active facilitators

Closely linked to facilitators feeling they needed to provide correct answers to participants was how they also felt they needed to be ‘active facilitators’ to have been successful. Being active facilitators included continually providing input to participants, leading sessions and talking, in essence positioning themselves as group leaders. Creating Futures, in particular, emphasized more individual work, often supported through individual journals, requiring facilitators to take a back seat and not guide the group:

Wanda (male): I feel like I am not contributing because they do lots of work than I just have to write up their ideas on a flip chart. Yes I don’t know maybe it’s my problem, I don’t know how, because there is lots of writing they must do and less of me than before, what can I report?

There was a clear sense amongst facilitators that if they were not at the front of the group sessions and guiding the group through an activity they were not working. On a daily basis facilitators were asked to complete short reports highlighting topics discussed in sessions and any challenges that emerged that needed to be dealt with in following sessions. On days when the sessions required facilitators to ‘sit back’ they felt they had little to say and they had not had a successful session:

Jabu (male): I feel like I am doing nothing because last time on the Stepping Stones there was a lot of writing and I would say, ‘ey my report, ey my report.’ Now I am doing nothing. I come with the two papers and then I write something and I say, ‘Ah, I am doing nothing now!’

In wanting to be active facilitators they implicitly positioned themselves as teachers in the group. This was also evident in how they moved towards a language of referring to group sessions as ‘classes’ inadvertently invoking the language of formal education, with its connotations of a teacher and compliant students [16, 18].

### ‘Achieving’ behaviour change

Facilitators’ final understanding of successful facilitation was in ‘achieving’ behaviour change amongst participants. Freire’s own work recognizes that behaviour change is a process that is unlikely to happen quickly and participants often move ‘forwards and backwards’ as they attempt to change [9, 8]. Moreover, research suggests participatory behaviour change interventions do not work quickly, nor do they work for all. Yet this complex understanding of behaviour change had not been clearly articulated to facilitators, who held an understanding of behaviour change that was more closely linked to Freire’s notion of banking education—once someone was taught something they should act on it [8]. As such, facilitators often discussed how participants had failed to modify their behaviour despite the fact that they had discussed it in sessions. Thandi describes her frustration of how some of the women in her group were still not using condoms consistently despite the fact that they had repeatedly discussed condom use and undertaken a number of role-plays in sessions:

Thandi (female): In my group most of them they still don’t use condoms the correct way like we’ve talked about it, I don’t know!

Similarly Jabu also described how his group of young men also continued not to use condoms:

Jabu (male): Another thing that I don’t know, if it’s bad or something we learn about condoms this week on Thursday guys, when you do this, use protection. A participant came the next Thursday with a problem of the ‘drop’ [gonorrhoea] or something, and if you tell the guys “I always tell you use a condom because there are consequences with this”, they reply, “you gave us one condom, I used one condom and then the next round I use I took out the condom and I continue, but I was hurting”. I tell them we must make it a fashion to carry condoms in our bags [giggles].

Again, facilitators rapidly moved away from a focus on supporting young people to reflect on their behaviours and understand the broader social contexts and constraints in which young people lived towards individualized understandings of behaviour and behaviour change. Through a focus on individual behaviour facilitators missed an opportunity to open up a discussion on the social roots of behaviour (in this case condom use).

## Discussion

The facilitators held multiple understandings of what constituted successful facilitation in the delivery of Stepping Stones and Creating Futures. These differing conceptualizations of success had implications for whether dialogue would have been promoted and in turn lead to critical thinking amongst participants—the key theoretical underpinning of behaviour change in participatory interventions such as this [5, 8, 18].

Importantly, facilitators had internalized the importance of dialogue in their everyday practices of facilitation and this became part of their identity of good facilitators and their daily practice. This contrasts with other studies that suggest that the challenges facilitators face in delivering participatory interventions are overwhelming and they move rapidly towards didactic approaches (e.g. Ref. 16). It may have been because the project provided 5 weeks of training, plus ongoing close mentorship and support through a senior trainer. Through this model of training and support the facilitators came to start to understand not only the intervention, but the theoretical reasons behind the intervention.

Yet in understanding how facilitators constructed successful sessions it is necessary to move beyond a narrow focus on training and support to understand how wider contextual factors shaped their understanding. Three wider factors were important: (i) individual versus contextual factors of behaviour and behaviour change; (ii) experiences of education in South Africa and (iii) a tension between the intervention and the research project within which it was embedded.

The first contextual factor was how facilitators understood the drivers of young people’s behaviour, either seeing them as autonomous, rational individuals or as constrained by wider social and economic factors that shaped and limited their behaviours. This tension about people’s autonomy, essentially a debate around structure versus agency, is at the heart of modern social science and is framed by ideological viewpoints about human nature and health promotion [7, 34]. Popular understandings of human behaviour position individuals as being rational and able to respond with autonomy to new information in ways that will promote their own health and wellbeing [34]. However, the intervention and theory of behaviour change explicitly located young people’s behaviour as occurring because of the wide economic and social constraints they faced in their lives and how these factors constrained the potential for change [5, 29, 35].

Facilitators were torn between these two conceptualizations of human behaviour. The training they received as facilitators supported them to recognize the constraints young people faced and to work with young people to help them recognize these. Indeed at times facilitators were acutely aware of the constraints shaping young people’s decisions and choices, particularly around attending sessions. Yet when facilitators felt resistance by participants and were challenged they rapidly slipped back into individualized understandings of behaviour, blaming young people for a failure to change their behaviour, or not arrive to sessions on time, in so doing quickly foregrounding participants’ agency. While with a different emphasis, this broadly parallels what Campbell and MacPhail [16] described in their study where facilitators were unwilling to focus on social drivers of HIV-vulnerability and rapidly moved towards dominant biological understandings and as such moved towards individualized ideas of causation.

The second contextual factor shaping facilitators’ understandings of success was their experience of education and work in South Africa. In short, facilitators’ experiences of education and work had been in hierarchical and didactic situations and they drew on these repertoires in their facilitation, which contrasted sharply with an emphasis on participation and dialogue. Much has been written about the didactic nature of the education system and the challenges of delivering participatory interventions in these contexts [13, 16, 18]. Similarly the majority of work experiences in South Africa—and globally—reflect similar notions with clear hierarchies of power. As such, facilitators quickly started to draw on their institutional power [36] of being facilitators who were imbued with knowledge and status that participants did not have. Furthermore, as facilitators held a slightly higher economic and social position compared to participants—all but one came from a slightly higher socio-economic position in terms of where they lived and inadvertently demonstrated this in clothes and how they spoke—this subtly reinforced their position of power.

In addition education and work approaches in South Africa are clearly structured around definable and measurable outcomes and facilitators wanting to demonstrate their commitment to work sought clear markers of this throughout the intervention. As such they tended to favour clearly measurable markers such as attendance and active facilitation, all of which could be readily assessed quantitatively, rather than the intangible outcomes of dialogue and critical thinking and change over a long period that were difficult to demonstrate and measure. A significant body of research has identified how in lay counselling those delivering interventions quickly move towards working on readily measurable aspects of work, rather than focusing on softer, less easily definable outcomes [37, 38]. This is not surprising in contexts of high levels of unemployment where facilitators were keen to demonstrate they were working in the hope of future employment, alongside the continual focus on quantifiable measures of work performance in previous work situations.

The final contextual factor shaping how facilitators constructed success in the delivery of the intervention was the tension between the requirements of a research project and of the participatory approach of the intervention. For the research project there was a significant focus by team members on two key concepts in research, fidelity—that is the intervention was delivered in the way intended—and ensuring participants attended the intervention to achieve ‘dosage’ [22, 39]. Failure to achieve dosage and fidelity undermines the potential for interventions to show effect. This was an ongoing concern during implementation and the research team were concerned about attendance and fidelity to the intervention [22]. To try and improve attendance the research team put in place structures to encourage facilitators to contact participants and follow up more often with those who had not attend a session, including small monetary rewards (R100/US$10) each week for the person with the highest rate of attendance. These very specific and measurable markers of success were certainly important in how facilitators started to understand success and contrasted sharply with the ‘softer’ and more intangible markers of successful participatory outcomes of ‘good’ facilitation which focuses on recognising the process is continually evolving and enabling this to happen [9].

The study had a number of limitations. There was a lack of diversity in research methodologies, potentially providing a narrow understanding of how the intervention was implemented. The composition of team meetings (with facilitators, research staff and the senior trainer) no doubt increased the unequal power dynamics inherent in the situation and led to facilitators seeking to portray their experiences in particular ways. However, despite these relationships of power, facilitators did appear to be open in their responses.

## Conclusion

Behavioural approaches to health promotion, particularly in the context of HIV- and IPV-prevention, continue to be dominated by participatory methodologies [3, 6]. These rely on the skill of facilitators to manage the complex process of supporting dialogue amongst participants, which will generate critical thinking and behaviour change [7, 14]. However, as the case study of the Stepping Stones and Creating Futures demonstrates, while critically important these skills are difficult for people to learn and operationalize, particularly in contexts where people’s experiences of education have been didactic and where there are multiple factors that shape how facilitators deliver interventions. What possible strategies may be of use in supporting the translation of facilitation skills to large-scale delivery of participatory interventions?

Training and ongoing support are central to the effective delivery of participatory interventions at scale [12]. Yet despite 5 weeks of training—significantly more than in many other interventions—this did not overcome all the challenges, although it did address some. It may be important then that during the training facilitators have discussions on the different theoretical models of education, specifically between education as banking and education as critical consciousness [8], which underpin interventions. This would need to include engaging with facilitators around understandings of human behaviour (agency versus structure). Through explicitly highlighting the theoretical underpinnings of the intervention, facilitators may start to be able to locate their own practice within a wider system and come to understand the factors shaping their own facilitation. Essentially, what is required is for facilitators themselves to go through a process of reflection, critical consciousness raising and change as outlined by Freire, which will enable them to understand how their own experiences of education, work and understandings of human behaviour inflect on their daily practice of facilitation and in turn work to develop alternative models.

In addition, the study suggests that facilitators require ongoing support and spaces to reflect on their practices of facilitation. Models such as reflective diaries and group discussions are important in creating these safe social spaces for reflection [40]. These spaces need to move beyond discussions of work-tasks towards supporting facilitators to strengthen participatory approaches. In particular, this may include role-modelling of reflection and dialogue, and ongoing self-critique around their own process of facilitation.

Yet, the assumption that interventions relying on participatory models of behaviour change will simply ‘work’ with enough training and support for facilitators, may be inappropriate. There are structural challenges to shifting approaches, rooted in histories of education and dominant conceptualizations of human agency, as well as on-going conflicts such as the management of work and the requirements of research projects. Indeed, it may simply be that the ideals of participatory methodologies cannot be reconciled in the context of research projects, or time-bound donor funded interventions. Indeed, the idea that ‘transformative-participation’ is possible in such interventions has been widely questioned [41]. In reality, these will be ongoing tensions that programme implementers and researchers need to be aware of as they use participatory methodologies to effect behaviour change and not ones that can be removed simply by training and support; however, continued training and support will lead to better outcomes.

The emphasis on strengthening and extending training and support for facilitators is at odds with the global push for task-shifting and delivering interventions at low-cost. However, the continued limited impacts of behavioural HIV-prevention and IPV-prevention interventions, will continue without developing models of training and support that enable participatory behaviour change interventions to be delivered at scale, while retaining their underlying theory of change.

## Funding

R.J., N.J.-S. and S.W. were supported by the MRC of South Africa and received funding from DFID. A.G. and L.W. received funding from DFID. A.G. and S.W. received funding from SIDA. The project was also funded by SIDA, Norad, the Joint Gender Fund (South Africa) and the South African Medical Research Council (MRC). This document is an output from the What Works to Prevent Violence: a Global Programme, which is funded by UK Aid from the UK Department for International Development (DFID) for the benefit of developing countries. However, the views expressed and information contained in it are not necessarily those of or endorsed by DFID, which can accept no responsibility for such views or information or for any reliance placed on them. 

## Conflict of interest statement

None declared.
